# Creative mind links art and science

**DOI:** 10.3325/cmj.2016.57.87

**Published:** 2016-04

**Authors:** Sandor G. Vari

**Affiliations:** International Research and Innovation in Medicine Program, Cedars-Sinai Medical Center, Los Angeles, CA, USA & Association for Regional Cooperation in the Fields of Health, Science and Technology (RECOOP HST Association), Debrecen, Hungary *vari@cshs.org*

“There is only one difference between a madman and me. I am not mad.”– Salvador Dali

Do we need to be crazy to be creative? Behavioral and brain researchers have found a number of strong, although indirect, ties between an original mind and a troubled one. We could find several publications accepting that mental illness and creativity are linked. Nevertheless, I completely agree with Scott Barry Kaufman, who believed that mental illness was neither necessary nor sufficient for creativity, and that mainly experience and intellect predicted creative achievement in the arts and sciences (1).

For Steve Jobs, one of the most famous brilliant persons, the white was not white enough for the iPhone design. He was “obsessive” until the last minute. When he was dying of cancer, he demanded that his oxygen mask be redesigned. Does it mean Steve Job was extremely obsessed (2)? In my view, Steve Jobs was a perfectionist, and he pushed himself beyond the normal limits to achieve his goals, leaving us all impressed by his accomplishments. He motivates me in my research and research management. In the last two decades I have learned that the most important element of research and research networking is creative thinking (3).

James Gimzewski, who pioneered in research on electrical contacts with single atoms, molecules, and light emission using scanning tunneling microscopy, believes that “Traditionally, scientists have been trained to be reductionists – reducing complex data and phenomena to simple terms. But that approach has reached its limits” (4). New research approaches and concepts are not the expansions of existing knowledge; they leave the comfort zone of “chamber science,” changing the dynamic of science and integrating individual disciplinary models into multidisciplinary research (3).

Arts and sciences shape culture, and culture shapes how individuals judge the world. The new generation communicates on a fast-track what they consider right and wrong or suitable and unsuitable via Facebook, Twitter, or other social media. Researchers are doing the same on ResearchGate and other academic social networks (5,6). Although artists have no giant social networks, they can easily connect also on the net. Using our browsers, we could find all the information, we just have to use the right combination of keywords. The human-machine interaction, the web-based information gathering, and the internet connectivity will help to access a vast amount of information that could initiate creative thinking, but never will they replace the face to face interaction to willingly explore different points of view.

Hippocratic Oath is the best known medical text worldwide. At the same time, the modern physicians using sophisticated machinery throughout the diagnostic process are missing Hippocratic “bedside medicine” and are less and less using human senses in medical diagnosis (7). If we use our imaginations when looking at a piece of art, we can also describe it using all five senses: hearing, sight, touch, smell, and taste. Artist and scientists have common “tools” – the five senses as traditionally recognized methods of perception (8).

Arts and sciences are sometimes described as separate human activities, mismatched and contradicting each other. From my point of view, art and science are not so disparate. They both require careful observation, intuition, inspiration, passion, dedication, and discipline, and both can inspire each other. In science and art, the most essential thing is to create something genuine based on the free association of thoughts. Science has to be more guided toward a well-defined goal summarized in the hypothesis, while art is more spontaneous, logically unconstrained, using an undirected association of ideas, emotions, and feelings.

The RECOOP HST Association, together with Korányi Frigyes College for Advanced Studies, Semmelweis University, Budapest, Hungary (*http://harsfa.semmelweis.hu/recoop-fkcas-lsiiaw.php*), announced the first annual Art and Sciences Competition. The main goal of the RECOOP Annual Art and Sciences Competition is to initiate a creative working relation between RECOOP young scientists and artists to create artworks from life science and medical images and communicate the beauty of the living organism from cytoplasmic organelles through “little organs” that are suspended in the cytoplasm of the cell to nanoparticles used in medicine. RECOOP HST Association with the involvement of young artists inspires young scientists and clinical researchers for creative thinking, and creates a platform for multicenter, transdisciplinary, and transnational collaborations.
